# Mental health disorders among children with special health needs: A population-based cohort study using linked administrative data from Manitoba, Canada

**DOI:** 10.1371/journal.pone.0326672

**Published:** 2025-06-25

**Authors:** Jennifer E. Enns, Caroline Reid-Westoby, Gilles R. Detillieux, Marni Brownell, Nathan C. Nickel, Anne Gadermann, Astrid Guttmann, Teresa Bennett, Eric Duku, Barry Forer, Monique Gagné Petteni, Katholiki Georgiades, Martin Guhn, Ana Hanlon-Dearman, Brenda T. Poon, Magdalena Janus

**Affiliations:** 1 Manitoba Centre for Health Policy, Department of Community Health Sciences, Max Rady College of Medicine, Rady Faculty of Health Sciences, University of Manitoba, Winnipeg, Manitoba, Canada; 2 Offord Centre for Child Studies, Department of Psychiatry and Behavioural Neurosciences, McMaster University, Hamilton, Ontario, Canada; 3 Human Early Learning Partnership, School of Population and Public Health, University of British Columbia, Vancouver, British Columbia, Canada; 4 Centre for Advancing Health Outcomes, Providence Health Care Research Institute, Vancouver, British Columbia, Canada; 5 The Hospital for Sick Children, Toronto, Ontario, Canada; 6 The Edwin SH Leong Centre for Healthy Children, University of Toronto, Toronto, Ontario, Canada; 7 ICES, Toronto, Ontario, Canada; 8 Department of Pediatrics, Institute of Health Policy, Management and Evaluation, Dalla Lana School of Public Health, University of Toronto, Toronto, Ontario, Canada; 9 Department of Pediatrics and Child Health, Max Rady College of Medicine, Rady Faculty of Health Sciences, University of Manitoba, Winnipeg, Manitoba, Canada; Karolinska Institutet, SWEDEN

## Abstract

**Objective:**

An estimated 15–22% of Canadian kindergarten-age children have a special health need (SHN), defined as a clinical diagnosis, a functional need requiring special accommodation at school, or a health condition leading to increased needs. Children with SHN may be more likely to experience mental health disorders than their peers without SHN, placing them at risk for further health and academic challenges. Our objective was to determine the odds of children with SHN identified in kindergarten being diagnosed with a mental health disorder by age 16.

**Methods:**

In this retrospective cohort study using population-based, linked administrative data, we identified children with SHN born 1995–2020 in Manitoba, Canada, and enrolled in kindergarten from 2006–2011. The SHN designation is derived from the Early Development Instrument. We measured prevalence of common childhood mental health disorders (ADHD, mood/anxiety disorders, conduct disorders) in children with SHN to age 16. Using binary logistic regressions, we calculated crude odds ratios (OR) for children with vs. without SHN being diagnosed with a mental health disorder, then adjusted for age, sex, and neighbourhood-level income.

**Results:**

Among 42,766 children, 13.8% had a SHN designation in kindergarten. Among these, 41.0% were diagnosed with a mental health disorder by age 16. The odds of a mental health diagnosis by SHN category were: special needs designation in kindergarten (OR 1.75, 95%CI 1.53–2.01); learning impairment (OR 1.61, 95%CI 1.39–1.86); behavioural impairment (OR 3.27, 95%CI 2.87–3.72); and emotional impairment (OR 2.01, 95%CI 1.75–2.32). Children with SHN (vs. none) had higher odds of a mental health disorder if they had 1 + impairment (OR 1.67, 95%CI 1.50–1.85). Adjusting for sociodemographic characteristics did not change the estimates.

**Conclusions:**

The study highlights important kindergarten predictors of future mental health disorders in children, which should be used to inform preventive and supportive strategies for children with SHN and help generate wider mental health supports in schools.

## Introduction

In Canada, an estimated 15–22% of children in kindergarten have special health needs (SHN), defined as either a professionally-identified clinical diagnosis, a functional need requiring classroom accommodation, or one or more health conditions generating needs greater than those expected of typically-developing individuals, regardless of identification or diagnosis [1]. The SHN designation encompasses a wide range of conditions including neurodevelopmental disorders (e.g., Autism Spectrum Disorder, Attention Deficit Hyperactivity Disorder [ADHD]), physical and sensory disorders (e.g., cerebral palsy, vision and/or hearing disorders), intellectual disabilities, and learning impairments [2].

Children with SHN face many potential challenges in their development compared to their peers without SHN, including a higher vulnerability to emotional and behavioural problems [3] and a higher likelihood of experiencing mental health disorders [4–9]. Much of the known evidence comes from research on intellectual disabilities: a study in the UK showed 36% of adolescents with intellectual disabilities live with mental health disorders vs. 8% of those without [10], and a systematic review estimated the comorbidity of intellectual disabilities and mental health disorders to be 30–50% [9]. A meta-analysis of 19 studies of mental health disorders in children and youth with intellectual disabilities found a similar prevalence of 38–49%, which was higher than in children without disabilities [11]. But despite the higher prevalence of mental health disorders in children with a broad range of disabilities, they experience more barriers to receiving a diagnosis and accessing support services [3,11].

Children and youth with SHN are also disproportionately exposed to inequities associated with socioeconomic status (SES), such as low family income, inadequate housing conditions, or requiring income assistance [12]. Moreover, the neighbourhood SES gradient in the development of young children with SHN is as pronounced as among children without SHN [13]. Our recent study shows that Canadian children with SHN are more likely to live in lower income neighborhoods, with the strength of this association varying by their province of residence [14,15]. Whether or not children are recent immigrants also plays a role [16,17], suggesting immigrant children with developmental disabilities are less likely than non-immigrant children to have access to the services they require [18]. Another international review concluded foreign-born children with special health needs are at particular risk of not having adequate access to health professionals [17].

Aside from these studies, evidence on the association between SHN and mental health disorders remains limited, particularly in Canada, and the relationship between SHN and mental health has not been previously investigated in a longitudinal population‐based study that accounts for key social determinants of health. The current study examines whether the SHN designation and its sub-categories, measures of children’s functioning in the classroom, are associated with a later diagnosis of a mental health disorder.

## Methods

### Study setting and design

Manitoba is a Canadian province with a population of 1.4M. Its demographic and educational profiles align closely with national averages [19,20], as such, a population-based study will be fairly generalizable to Canada as a whole. For this retrospective cohort study, we linked data from the Early Development Instrument (EDI), a population-level, kindergarten teacher-completed questionnaire of children’s developmental health [21], to the individual-level administrative health data in the Manitoba Population Research Data Repository. In addition to data on child development in major developmental domains, the EDI collects information on child health (such as functional impairments), administrative information (such as being designated in the special needs category), and demographic information (such as age, sex at birth, postal code of residence, and first language spoken). We calculated the prevalence of mental health disorders among children with and without SHN in kindergarten and, using binary logistic regressions, estimated the odds of children with various types of SHN receiving a mental health disorder diagnosis by age 16.

### Data sources

The Manitoba Population Research Data Repository is housed at the Manitoba Centre for Health Policy, University of Manitoba [22]. It contains routinely collected administrative records of contacts with the health system, social services, the education system, and the justice system for virtually the entire population (>99.9%) of Manitoba. All records in the Repository are de-identified (names and addresses removed), but they are linkable at the individual and family levels to a central population registry by way of a scrambled numeric identifier (each person’s Personal Health Identification Number). The scrambled identifier is attached to each record by a third party before the records are transferred to the Repository. Through the central registry, records from various databases can then be linked across domains and over time to conduct cross-sectional and longitudinal studies. The databases are subject to extensive quality assessment to ensure standards for linkage accuracy, completeness, and other data quality metrics are met before they are used for research [23].

The specific databases used in this study were: the Manitoba Health Insurance Registry (birth date and biological sex); Statistics Canada public use data (postal codes used to develop income quintiles); Hospital Discharge Abstracts (mental health disorders); Medical Claims/Medical Services (mental health disorders); the Drug Program Information Network (mental health disorders); Immigration, Refugees, and Citizenship Canada Permanent Resident Database (immigration status); and the EDI database (SHN designations). Our team had access to the de-identified databases for this study starting October 27, 2023.

### Cohort

**Fig 1:**
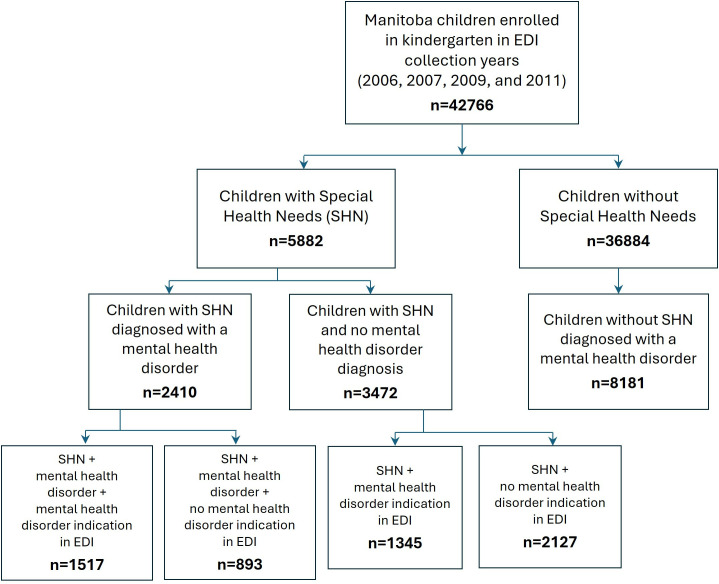
Cohort Construction. EDI: Early Development Instrument; SHN: Special Health Needs.

A cohort development flowchart is shown in Fig 1. We first identified children born from 1995–2020 to align with the years for which EDI data were available. We excluded any children who did not have continuous health insurance coverage from September 1 of the year they turned 5 up to their 16^th^ birthday. Anyone who moved out of province or died during that time period was excluded from the cohort. We then narrowed the cohort to children enrolled in kindergarten in years the EDI was implemented in Manitoba (2006, 2007, 2009, and 2011). Although EDI collection also occurred in later years, limiting our study period to 2011 and earlier allowed us to follow the cohort up to age 16 (i.e., up to 2016 for the youngest children in the cohort) to assess mental health. We divided the cohort into those who did and did not have an SHN designation, and those who did and did not receive a mental health disorder diagnosis (see below for definitions). Those with the SHN designation were further subdivided into those who did or did not have a mental health disorder indication in their EDI records to determine whether this indicator resulted in a higher likelihood of a mental health disorder diagnosis than the SHN designation alone.

### Variables

#### Exposure.

Special health needs: A child was identified as having SHN if they met any of the following criteria based on the variables available in the EDI database: 1) school-district determined designation of special needs in kindergarten (one question, yes/no answers); 2) teacher-reported functional impairment, specifically physical, vision, hearing, speech, learning disability, behavioural, or emotional (one question per impairment type, yes/no answers); or 3) teacher-reported need for further assessment to include children who may have been identified as needing additional assistance in the classroom, but have not yet received a formal diagnosis or designation of special needs (one question, yes/no answers).

#### Covariates.

Demographic variables: Age, sex, and household income were measured descriptively and included as covariates in the regression models. Age and biological sex were obtained from the Manitoba Health Insurance Registry. Income quintiles, which are used as a proxy for household SES, were calculated from public use Canada Census files using dissemination area-level average household income. Average household incomes were ordered from lowest to highest income and grouped into five categories (quintiles). In the binary regression models, we compared the four lowest quintiles individually to quintile 5 (Q5, highest income). Immigrant to Manitoba: A child was determined to be an immigrant to Manitoba if they were linked to a landing record in the Immigration, Refugees and Citizenship Canada Permanent Resident Database, indicating they were born outside of Canada.

#### Outcome measures.

Mental health disorder diagnoses: The variables used to define physician- or nurse practitioner-diagnosed mental health disorders came from the physician billing claims, hospital discharge data, and/or prescription drug data. We examined mood/anxiety disorders, ADHD, conduct disorders, and ‘any’ of these three types of mental health disorders in the administrative health data. The disorder definitions were developed from ICD-9 CM and ICD-10 CA diagnostic codes through collaboration with expert mental health clinicians and clinician researchers and have been used extensively in previous investigations of Manitoba children’s mental health [24–26]. The detailed definitions for each disorder, including ICD and ATC codes, are provided in S1 Table.

### Subgroup analysis

Mental health disorder indication – a teacher-indicated flag in the EDI dataset: Since some of the criteria to receive a SHN designation include aspects of mental illness, and since one of the developmental domains of the EDI, the emotional maturity domain, has subdomains which reflect symptoms of aggression, hyperactivity, and anxiety [27], we conducted a subgroup analysis to examine an additional flag in the EDI data that indicates a child may have an undiagnosed mental health disorder in kindergarten. A child was considered to have a mental health disorder indication if they met the following criteria in the EDI data: 1) they had a teacher-reported behavioural or emotional impairment; or 2) they met “none or very few” expectations in the subdomains of anxiety, aggressive behaviour, or inattention and hyperactivity. This analysis allowed us to explore whether the mental health disorder indication flag could be used as a predictor of a mental health diagnosis in future work.

### Statistical analysis

All analyses were completed in SAS/STAT® software (V9.4) [28]. We generated descriptive counts and percentages of the sociodemographic characteristics and the prevalence of mental health disorders in children with and without SHN. We estimated the likelihood of a child with SHN being diagnosed with a mental health disorder, overall and by category of SHN, using binary logistic regression, generating odds ratios and 95% confidence intervals. Each SHN category was evaluated in its own regression model, comparing children with a mental health disorder diagnosis to those without, using both unadjusted and adjusted models. Adjusted models included neighbourhood-level income quintile, age (below mean at date of EDI completion), and sex at birth as categorical covariates. As a final check, we stratified the cohort bysex and compared the findings to those from the adjusted regression models to ensure that any confounding due to sex had been accounted for in those models.

### Ethics

The study received approval from the University of Manitoba Health Research Ethics Board (HS22691 H2019:110). The requirement for informed consent from individuals represented in the data was waived by the ethics review board because the study used secondary data that were de-identified by a third party before being transferred into the Repository. Strict measures were in place to protect the privacy of individuals in the study, and there was minimal risk that any individual could be identified at any stage of the research. The protocol for the study was also reviewed for privacy concerns by the Manitoba Government’s Health Information Privacy Committee (#2018/2019–70).

## Results

The study cohort development is shown in Fig 1. There were 42,766 children enrolled in kindergarten during the study years in which the EDI was collected in Manitoba. Among these children, 5,882 (13.8%) had a SHN designation in their EDI record, and among the children with SHN, 2,410 (41.0%) were diagnosed with a mental health disorder by the time they were 16 years old. Among the 36,884 children without SHN, 8,181 (22.2%) were diagnosed with a mental health disorder by the time they were 16 years old. We further classified children by whether they had a mental health disorder indication in their EDI record to determine whether this designation contributed to the likelihood of a mental health disorder diagnosis. The results of these analyses are not presented or discussed further in the main manuscript, but the relevant data are shown in S2–S5 Tables.

The cohort’s sociodemographic characteristics are presented in Table 1. Among children with SHN, 65.4% were male and the average age at first mental health disorder diagnosis was 11.76 years; 3.2% were immigrants to Manitoba, and an income gradient was evident, with more children from lower-income families having a designation of SHN. Among children without SHN, 48.2% were male and the average age at first mental health disorder diagnosis was 12.55 years; 5.4% were immigrants, and although there was an income gradient in the same direction, it was less pronounced than in the SHN group.

**Table 1:** Sociodemographic Characteristics of Manitoba Children at School Entry. Manitoba children in kindergarten in EDI collection years 2006, 2007, 2009, and 2011.

	Total Cohort		Special Health Needs				No Special Health Needs			
			All		With Mental Health Disorder Diagnosis		All		With Mental Health Disorder Diagnosis	
	N	%	N	%	N	%	N	%	N	%
**Total Counts and %**	42766	100	5882	100	2410	100	36884	100	8181	100
**Sex**										
** Male**	21609	50.5	3848	65.4	1654	68.6	17761	48.2	3755	45.9
**Income Quintiles**										
** Q1 (lowest)**	9935	23.2	1747	29.7	768	31.9	8188	22.2	2031	24.8
** Q2**	8693	20.3	1195	20.3	496	20.9	7498	20.3	1713	20.9
** Q3**	8434	19.7	1089	18.5	453	18.8	7345	19.9	1546	18.9
** Q4**	8326	19.5	1042	17.7	386	16.0	7284	19.8	1511	18.5
** Q5 (highest)**	7190	16.8	781	13.3	289	12.0	6409	17.4	1347	16.5
** Income Quintile Not Available**	188	0.4	28	0.5	18	0.8	160	0.4	33	0.4
**Immigrant to Manitoba**	2163	5.1	188	3.2	43	1.78	1975	5.4	203	2.5
	**Mean**	**SD**	**Mean**	**SD**	**Mean**	**SD**	**Mean**	**SD**	**Mean**	**SD**
**Age at EDI Completion**	5.69	0.30	5.69	0.33	5.70	0.34	5.69	0.29	5.67	0.29
**Age at First Mental Health Disorder Diagnosis**	12.42	2.99	11.76	3.16	11.76	3.16	12.55	2.94	12.55	2.94
**Age at First Mental Health Disorder Diagnosis after School Entry**	12.50	2.88	11.83	3.07	11.83	3.07	12.63	2.82	12.63	2.82

EDI: Early Development Instrument; SD: standard deviation. ‘Mental health disorder diagnosis’ includes mood or anxiety disorder, ADHD, and/or conduct disorder. ICD codes and algorithms used to determine diagnoses are presented in S1 Table.

In Table 2, we present the different categories of impairments by which children with SHN were classified. Among all children with SHN, the most common were speech impairments (34.3%), followed by behavioural impairments (21.8%). Nearly half (46.1%) of children with SHN had more than one type of impairment and just over two thirds (66.8%) were considered in need of further assessment. Among children with SHN who were later diagnosed with a mental health disorder, a notably higher proportion had an emotional (21.4% vs. 15.8%) or behavioural (33.7% vs. 21.8%) impairment; 53.6% had more than one type of impairment and 70.7% were in need of further assessment.

**Table 2:** Prevalence of Special Health Needs Categories among Manitoba Children. Manitoba children in kindergarten in 2006, 2007, 2009, and 2011.

	Children with Special Health Needs			
	All		With Mental Health Disorder Diagnosis	
	N	%	N	%
**Total Counts and %**	5882	100	2410	100
**Special Needs in Kindergarten**	1000	17.0	524	21.7
**Physical Impairment**	211	3.6	83	3.4
**Vision Impairment**	211	3.6	78	3.2
**Hearing Impairment**	225	3.8	79	3.3
**Learning Impairment**	849	14.4	433	18.0
**Speech Impairment**	2017	34.3	698	29.0
**Behavioural Impairment**	1281	21.8	813	33.7
**Emotional Impairment**	930	15.8	516	21.4
**Teacher-Reported Need for Further Assessment**	3930	66.8	1704	70.7
**2 or more of these categories**	2712	46.1	1291	53.6

Columns do not total 100% because children may be in more than one category.

Table 3 shows the prevalence of common types of mental health disorders diagnosed in children aged 0–16. Forty-one percent of children with SHN were diagnosed with at least one mental health disorder, the most common of which were mood/anxiety disorders (18.0%) and ADHD (30.8%); among children without SHN, the proportion of children with at least one mental health disorder was 22.2% (14.2% mood/anxiety and 10.7% ADHD).

**Table 3:** Prevalence of Mental Health Disorders among Manitoba Children with and without Special Health Needs. Manitoba children in kindergarten in 2006, 2007, 2009, and 2011.

		Total Cohort		Special Health Needs		No Special Health Needs	
		N	%	N	%	N	%
**Total Counts and %**		42766	100	5882	100	36884	100
**Diagnosed from Age 0–16 Years**	**Mood/Anxiety Disorder**	6285	14.7	1059	18.0	5226	14.2
	**ADHD**	5743	13.4	1814	30.8	3929	10.7
	**Conduct Disorder**	2026	4.7	588	10.0	1438	3.9
	**Any Mental Health Disorder**	10591	24.8	2410	41.0	8181	22.2

Only 39 children (less than 0.4%) were diagnosed with a mental health disorder before school entry, so for simplicity’s sake, all children diagnosed from ages 0–16 years as a group. ‘Any mental health disorder’ includes mood or anxiety disorder, ADHD, and/or conduct disorder. ICD codes and algorithms used to determine diagnoses are presented in S1 Table.

**Fig 2:**
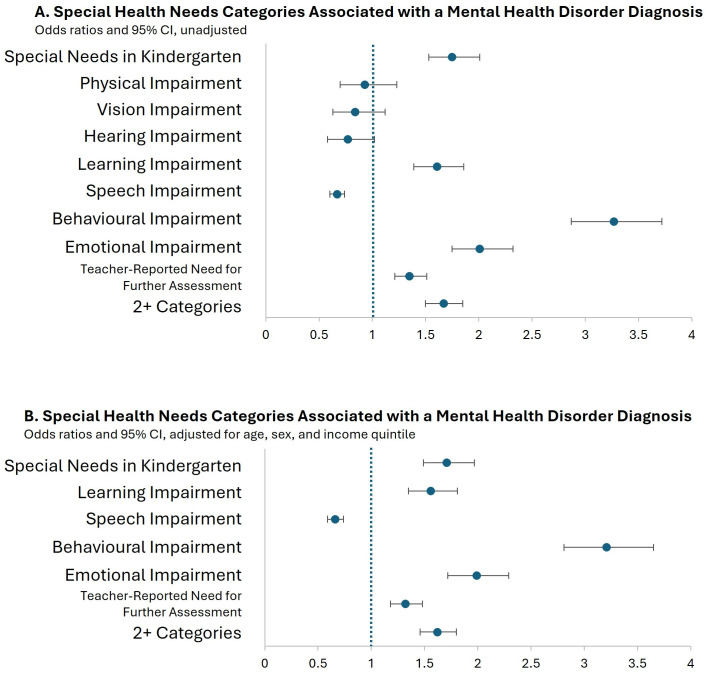
Special Health Needs Categories Associated with a Mental Health Disorder Diagnosis in Manitoba Children with Special Health Needs identified in Kindergarten in 2006, 2007, 2009, and 2011. A. Unadjusted odds ratios and 95% CI. B. Odds ratios and 95% CI adjusted for age, sex, and income quintile.

The association between being designated in one or more SHN categories at kindergarten and being diagnosed with a mental health disorder by age 16 is presented as odds ratios (OR) with 95% confidence intervals (95% CI) in Fig 2A (unadjusted) and 2B (adjusted for age, sex, and neighbourhood-level income). Number estimates for all data points in Fig 2 are available in S6 Table. In the unadjusted results (Fig 2A), the odds of being diagnosed with a mental health disorder for children in the various SHN categories compared to children without SHN were: special needs designation in kindergarten (OR 1.75, 95% CI 1.53–2.01), learning impairment (OR 1.61, 95% CI 1.39–1.86), behavioural impairment (OR 3.27, 95% CI 2.87–3.72), and emotional impairment (OR 2.01, 95% CI 1.75–2.32). Children with SHN also had higher odds of a mental health disorder diagnosis if they had more than one type of impairment (OR 1.67, 95% CI 1.50–1.85) or if they were flagged in the EDI as needing further assessment (OR 1.35, 95% CI 1.21–1.51). Children who had a speech impairment had lower odds of being diagnosed with a mental health disorder than children without SHN (OR 0.67, 95% CI 0.60–0.74). The adjusted estimates were very similar to the unadjusted ones: special needs in kindergarten (OR 1.71, 95% CI 1.49–1.97), learning impairment (OR 1.56, 95% CI 1.37–1.84), speech impairment (OR 0.66, 95% CI 0.59–0.74), behavioural impairment (OR 3.21, 95% CI 2.86–3.72), emotional impairment (OR 1.99, 95% CI 1.74–2.33), need for further assessment (OR 1.32, 95% CI 1.19–1.50), and more than one type of impairment (OR 1.62, 95% CI 1.49–1.84), indicating that the adjustment variables we selected had little impact on whether a child with SHN was more likely than a child without SHN to be diagnosed with a mental health disorder. There were no differences between the sex-stratified results and the adjusted estimates for any of the outcomes (data not shown).

## Discussion

Our study demonstrates that the prevalence of common childhood mental health disorders among Manitoba children is nearly twice as high in children with SHN (41%) than without SHN (22%) up to age 16. Among children with SHN who were diagnosed with a mental health disorder, a notably higher proportion had a behavioural (33.7% vs. 21.8%) or emotional (21.4% vs. 15.8%) impairment in kindergarten than those without a mental health disorder. Compared to children without SHN, children in several specific SHN categories had substantially higher odds of being diagnosed with any of the mental health disorders investigated here by age 16, e.g., 1.6 times higher odds with a learning impairment, 3.3 times higher odds with a behavioural impairment, and 2.0 times higher odds with an emotional impairment. Taken together, these results reveal a strong association between SHN designation in kindergarten and a mental health disorder diagnosis by age 16. In particular, the link between mental health disorder diagnosis and behavioural and/or emotional impairments highlights these SHN categories as potential early predictors of mental health concerns later in childhood or adolescence.

As a group, children with SHN identified in kindergarten underachieve compared to their typically-developing peers [29–31] and are more likely to have emotional and behavioural problems [3]. Although indirect evidence suggests children with SHN are also more likely to be diagnosed with a mental health disorder than those without SHN [4–9], this is the first population‐wide study to address this question by examining mental health disorders together with intersecting social determinants of health. Our study confirms a growing prevalence of physician-diagnosed mental health disorders among children in Canada. A previous Manitoba study reported an increase in the prevalence of mental illness from 12.5% in 2006–2009 to 14% in 2010–2013 among children aged 0–19 years [26]. Our estimate of 24.8% among all children aged 0–16 years may reflect increasing awareness of mental health concerns in the general public and amongst diagnosticians and may also be higher because of our focus on the most common childhood mental health concerns. Our findings also demonstrate the disproportionally higher prevalence of mental health disorders in Manitoba children with SHN up to age 16 (41%), which is similar to that reported in countries like the UK and USA [9–11]. We found that the prevalence of ADHD and conduct disorders was 2–3 times higher among children with SHN than without SHN, and the odds of being diagnosed with any mental health disorder was highest among children with emotional and behavioural impairments. None of the above-cited studies examine either SHN type (or intellectual disability type) nor do they look at specific mental health disorders to the same degree of granularity as our study; thus, our findings are an important and novel contribution to the literature. Our results also revealed children with speech and language disorders in kindergarten were less likely to be diagnosed with mental health disorders than children without SHN. The underlying reason for this difference remains unclear, and in fact, the results lie somewhat counter to previous reports [32,33], requiring further investigation. Surprisingly, neighbourhood-level income did not contribute to the association between SHN and mental health disorders in Manitoba. Our previous research points to the relevance of socioeconomic factors to various child development outcomes [34] and identifies nuances of different mental health diagnoses [35]. Together, these findings suggest SHN status on its own is a powerful predictor of the risks to mental health.

The study has a few important limitations warranting mention. EDI data are collected in publicly funded schools only; children at private and some First Nations schools are excluded. As well, EDI data collection in Manitoba occurred only every other year during the study period. These realities may limit the generalizability of our findings to some degree. Using administrative data to define mental health disorders means only children who have been seen by a provider and received a diagnosis or prescription are in the Repository. The Repository contains primary care, hospital, and pharmacy records, but mental health services provided by school counselors, psychologists, and social workers are not available. It is likely that our findings underestimate the true prevalence of mental health disorders; however, this only strengthens our confidence in the significant associations observed. Finally, we considered ADHD diagnosis a mental health disorder (even though a more accurate definition is a “brain disorder” [36]), as this is how it has been operationalized in mental disorder classifications historically and at the time that children and youth in the cohort were diagnosed.

## Conclusions

This longitudinal study of mental health disorders among children with SHN identified in kindergarten highlights a substantial difference between children with and without SHN. It brings attention to children’s mental health challenges by helping to identify early indicators and factors associated with mental health disorders among children with SHN that could be used to initiate wider mental health supports in schools. Since the SHN designation is a variable easily available to educators and health providers, our study has immediate relevance in suggesting the need to monitor children with this designation for potential mental health concerns.

## Supporting information

S1 Table International Classification of Disease diagnostic codes, Anatomical Therapeutic Chemical codes, and healthcare use algorithms used to define mental health disorders in children in the administrative data at the Manitoba Centre for Health Policy. (DOCX)

S2 Table Sociodemographic characteristics of the cohort at school entry. Manitoba children in kindergarten in EDI collection years 2006, 2007, 2009, and 2011.
(DOCX)

S3 Table Prevalence of mental health disorders among children with special health needs and a mental health disorder indication in their EDI Record. Manitoba children in kindergarten in 2006, 2007, 2009, and 2011.
(DOCX)

S4 Table Prevalence of special health needs categories. Manitoba children in kindergarten in 2006, 2007, 2009, and 2011.
(DOCX)

S5 Table Relationship between special health needs and receiving a mental health disorder diagnosis. Manitoba children with a mental health disorder indication in their EDI record in 2006, 2007, 2009, and 2011. Odds ratios and 95% confidence intervals.
(DOCX)

S6 Table Association between being designated special health needs and receiving a mental health disorder diagnosis by age 16 years. Manitoba children in 2006, 2007, 2009, and 2011. Odds ratios and 95% confidence intervals.
(DOCX)
