# Supplementary material for: Mental health disorders among children with special health needs: A population-based cohort study using linked administrative data from Manitoba, Canada
Source: PLoS One. 2025 Jun 25;20(6):e0326672. doi: 10.1371/journal.pone.0326672 (PMC12194185; doi:10.1371/journal.pone.0326672)
Supplement: S1 Table — (DOCX) [file pone.0326672.s001.docx]

| **S1 Table. International Classification of Disease diagnostic codes, Anatomical Therapeutic Chemical codes, and healthcare use algorithms used to define mental health disorders in children in the administrative data at the Manitoba Centre for Health Policy.** | |
| --- | --- |
| **Mood/anxiety disorders** | - 1+ hospitalizations with diagnosis codes: ICD9 296.1-296.8, 300.0, 300.2, 300.3, 300.4, 300.7, 309 or 311; ICD10 F31-F33, F341, F38.0, F38.1, F40, F41.0, F41.1, F41.2, F41.3, F41.8, F41.9, F42, F43.1, F43.2, F43.8, F53.0, or F93.0, or - 1+ hospitalizations with diagnosis codes: ICD9 300; ICD10 F32, F341, F40, F41, F42, F44, F45.0, F45.1, F45.2, F48, F68.0 or F99 and one or more prescriptions for an antidepressant or mood stabilizer: ATC codes N05AN01, N05BA, N06A, N05BE01, or - 1+ physician visits with a diagnosis code: ICD9 296, 311, OR, - 1+ physician visits with a diagnosis code: ICD9 300 and one or more prescriptions for an antidepressant or mood stabilizer: ATC codes N05AN01, N05BA, N06A, N05BE01, or - 3+ physician visits with a diagnosis code: ICD9 300, 309. |
| **ADHD** | - 1+ hospitalizations with diagnosis of hyperkinetic syndrome (ICD-9-CM code 314 or ICD-10 code F90) in one fiscal year, or - 1+ physician claims with diagnosis of hyperkinetic syndrome (ICD-9-CM code 314) in one fiscal year, or - 2+ Rx for ADHD drugs in one fiscal year without a diagnosis in the same fiscal year of conduct disorder (312/F63, F91, F92), disturbance of emotions (313/F93, F94), cataplexy/narcolepsy (347/G47.4), or - 1 Rx for ADHD drugs in one fiscal year with diagnosis of hyperkinetic syndrome (ICD-9-CM code 314 or ICD-10 code F90) in the previous 3 years. |
| **Conduct disorders** | - One or more hospitalizations with diagnosis of conduct disorders ICD-9: 312, ICD-10: F91 (All F91 codes except F91.3 - oppositional disorder), or - One or more physician visits with a diagnosis of conduct disorders ICD-9: 312. |
| **Any mental health disorder** | In this study, we defined ‘any’ mental health disorder as one or more of the above three disorders. |
| These definitions were developed for the Mental Health of Manitoba’s Children report, released in 2016 (<http://mchp-appserv.cpe.umanitoba.ca/reference/MHKids_web_report.pdf>). | |
