# Supplementary material for: Mental health disorders among children with special health needs: A population-based cohort study using linked administrative data from Manitoba, Canada
Source: PLoS One. 2025 Jun 25;20(6):e0326672. doi: 10.1371/journal.pone.0326672 (PMC12194185; doi:10.1371/journal.pone.0326672)
Supplement: S2 Table — Manitoba children in kindergarten in EDI collection years 2006, 2007, 2009, and 2011. (DOCX) [file pone.0326672.s002.docx]

| **S2 Table. Sociodemographic characteristics of the cohort at school entry.**  **Manitoba children in kindergarten in EDI collection years 2006, 2007, 2009, and 2011.** | | | | | | | | |
| --- | --- | --- | --- | --- | --- | --- | --- | --- |
|  | **Children with Special Health Needs** | | **With Mental Health Disorder Diagnosis** | | **With Mental Health Disorder Indication** | | **No Mental Health Disorder Indication** | |
|  | **N** | **%** | **N** | **%** | **N** | **%** | **N** | **%** |
| **Total Counts and %** | 5882 | 100 | 2410 | 100 | 1517 | 100 | 893 | 100 |
| **Sex** |  |  |  |  |  |  |  |  |
| **Male** | 3848 | 65.4 | 1654 | 68.6 | 1123 | 74.0 | 531 | 59.5 |
| **Income** |  |  |  |  |  |  |  |  |
| **Q1 (lowest)** | 1747 | 29.7 | 768 | 31.8 | 503 | 33.2 | 265 | 29.7 |
| **Q2** | 1195 | 20.3 | 496 | 20.6 | 300 | 19.8 | 196 | 22.0 |
| **Q3** | 1089 | 18.5 | 453 | 18.8 | 279 | 18.4 | 174 | 19.5 |
| **Q4** | 1042 | 17.7 | 386 | 16.0 | 248 | 16.5 | 138 | 15.5 |
| **Q5 (highest)** | 781 | 13.3 | 289 | 12.0 | 174 | 11.5 | 115 | 12.9 |
| **Immigrant to Manitoba** | 188 | 3.2 | 43 | 1.78 | 23 | 1.5 | 20 | 2.2 |
|  | **Mean** | **SD** | **Mean** | **SD** | **Mean** | **SD** | **Mean** | **SD** |
| **Age at EDI Completion** | 5.69 | 0.33 | 5.70 | 0.34 | 5.69 | 0.34 | 5.69 | 0.32 |
| **Age at First Mental Health Disorder Diagnosis** | 11.76 | 3.16 | 11.76 | 3.16 | 11.63 | 3.14 | 11.92 | 3.17 |
| **Age at First Mental Health Disorder Diagnosis after School Entry** | 11.83 | 3.07 | 11.83 | 3.07 | 11.70 | 3.06 | 12.00 | 3.09 |
| EDI: Early Development Instrument; SD: standard deviation. ‘Mental health disorder diagnosis’ includes mood or anxiety disorder, ADHD, and/or conduct disorder. ICD codes and algorithms used to determine diagnoses are presented in S1 Table. | | | | | | | | |
