# Supplementary material for: Mental health disorders among children with special health needs: A population-based cohort study using linked administrative data from Manitoba, Canada
Source: PLoS One. 2025 Jun 25;20(6):e0326672. doi: 10.1371/journal.pone.0326672 (PMC12194185; doi:10.1371/journal.pone.0326672)
Supplement: S3 Table — Manitoba children in kindergarten in 2006, 2007, 2009, and 2011. (DOCX) [file pone.0326672.s003.docx]

| **S3 Table. Prevalence of mental health disorders among children with special health needs and a mental health disorder indication in their EDI Record. Manitoba children in kindergarten in 2006, 2007, 2009, and 2011.** | | | | | | | |
| --- | --- | --- | --- | --- | --- | --- | --- |
|  |  | **Children with Special Health Needs and a Mental Health Disorder Diagnosis** | | | | | |
|  |  | **All** | | **With Mental Health Disorder Indication** | | **Without Mental Health Disorder Indication** | |
|  |  | **N** | **%** | **N** | **%** | **N** | **%** |
| **Total Counts and %** |  | 2410 | 100 | 1517 | 100 | 893 | 100 |
| **Diagnosed from Age 0-16** | **Mood/Anxiety Disorder** | 1,059 | 43.9 | 584 | 38.5 | 475 | 53.2 |
|  | **ADHD** | 1,814 | 75.3 | 1264 | 83.3 | 550 | 61.6 |
|  | **Conduct Disorder** | 588 | 24.4 | 396 | 26.1 | 192 | 21.5 |
|  | **Any Mental Health Disorder** | 2410 | 100 | 1517 | 100 | 893 | 100 |
| ‘Any mental health disorder’ includes mood or anxiety disorder, ADHD, and/or conduct disorder. ICD codes and algorithms used to determine diagnoses are presented in S1 Table. | | | | | | | |
