# Supplementary material for: Mental health disorders among children with special health needs: A population-based cohort study using linked administrative data from Manitoba, Canada
Source: PLoS One. 2025 Jun 25;20(6):e0326672. doi: 10.1371/journal.pone.0326672 (PMC12194185; doi:10.1371/journal.pone.0326672)
Supplement: S4 Table — Manitoba children in kindergarten in 2006, 2007, 2009, and 2011. (DOCX) [file pone.0326672.s004.docx]

| **S4 Table. Prevalence of special health needs categories.**  **Manitoba children in kindergarten in 2006, 2007, 2009, and 2011.** | | | | |  | |
| --- | --- | --- | --- | --- | --- | --- |
|  | **Children with Special Health Needs and a Mental Health Disorder Diagnosis** | | | | | |
|  | **All** | | **With Mental Health Disorder Indication** | | **Without Mental Health Disorder Indication** | |
|  | **N** | **%** | **N** | **%** | **N** | **%** |
| **Total Counts and %** | 2410 | 100 | 1517 | 100 | 893 | 100 |
| **Special Needs** | 524 | 21.7 | 369 | 24.3 | 155 | 17.4 |
| **Physical Impairment** | 83 | 3.4 | 53 | 3.5 | 30 | 3.4 |
| **Vision Impairment** | 78 | 3.2 | 44 | 2.9 | 34 | 3.8 |
| **Hearing Impairment** | 79 | 3.3 | 40 | 2.6 | 39 | 4.4 |
| **Learning Impairment** | 433 | 18.0 | 319 | 21.0 | 114 | 12.8 |
| **Speech Impairment** | 698 | 29.0 | 354 | 23.3 | 344 | 38.5 |
| **Behavioural Impairment** | 813 | 33.7 | 813 | 53.6 | 0 | 0.0 |
| **Emotional Impairment** | 516 | 21.4 | 516 | 34.0 | 0 | 0.0 |
| **Teacher-Reported Need for Further Assessment** | 1704 | 70.7 | 1104 | 72.8 | 600 | 67.2 |
| **2 or more of these categories** | 1291 | 53.6 | 986 | 65.0 | 305 | 34.2 |
| Columns do not total 100% because children may be in more than one Special Health Needs category. | | | | |  | |
