# Supplementary material for: Mental health disorders among children with special health needs: A population-based cohort study using linked administrative data from Manitoba, Canada
Source: PLoS One. 2025 Jun 25;20(6):e0326672. doi: 10.1371/journal.pone.0326672 (PMC12194185; doi:10.1371/journal.pone.0326672)
Supplement: S5 Table — Manitoba children with a mental health disorder indication in their EDI record in 2006, 2007, 2009, and 2011. Odds ratios and 95% confidence intervals. (DOCX) [file pone.0326672.s005.docx]

| **S5 Table. Relationship between special health needs and receiving a mental health disorder diagnosis.**  **Manitoba children with a mental health disorder indication in their EDI record in 2006, 2007, 2009, and 2011.**  **Odds ratios and 95% confidence intervals.** | | | | | |
| --- | --- | --- | --- | --- | --- |
| **Unadjusted** | **N** | **%** | **Odds Ratio** | **95% CI** | **Pr > Chi-Square** |
| **Special Needs** | 369 | 24.3 | 1.92 | 1.58, 2.33 | <.0001 |
| **Physical Impairment** | 53 | 3.5 | 1.07 | 0.71, 1.61 | 0.7434 |
| **Vision Impairment** | 44 | 2.9 | 0.93 | 0.60, 1.42 | 0.7278 |
| **Hearing Impairment** | 40 | 2.6 | 0.93 | 0.59, 1.46 | 0.7569 |
| **Learning Impairment** | 319 | 21.0 | 1.66 | 1.36, 2.02 | <.0001 |
| **Speech Impairment** | 354 | 23.3 | 0.83 | 0.70, 0.99 | 0.0344 |
| **Behavioural Impairment** | 813 | 53.6 | 2.16 | 1.86, 2.52 | <.0001 |
| **Emotional Impairment** | 516 | 34.0 | 1.16 | 0.99, 1.36 | 0.0653 |
| **Teacher-Reported Need for Further Assessment** | 1104 | 72.8 | 1.43 | 1.22, 1.67 | <.0001 |
| **2+ Categories** | 986 | 65.0 | 1.78 | 1.54, 2.07 | <.0001 |
|  | | | | |  |
| **Adjusted*** | **N** | **%** | **Odds Ratio** | **95% CI** | **Pr > Chi-**  **Square** |
| **Special Needs** | 365 | 24.3 | 1.89 | 1.56, 2.30 | <.0001 |
| **Learning Impairment** | 314 | 20.9 | 1.63 | 1.33, 1.99 | <.0001 |
| **Speech Impairment** | 349 | 23.2 | 0.81 | 0.68, 0.96 | 0.0161 |
| **Behavioural Impairment** | 806 | 53.6 | 2.15 | 1.85, 2.50 | <.0001 |
| **Emotional Impairment** | 511 | 34.0 | 1.18 | 1.00, 1.38 | 0.046 |
| **Teacher-Reported Need for Further Assessment** | 1094 | 72.7 | 1.40 | 1.19, 1.64 | <.0001 |
| **2+ Categories** | 977 | 65.0 | 1.76 | 1.51, 2.04 | <.0001 |
| *Adjusted for age below mean at EDI assessment, sex and income quintile. | | | | | |
