# Supplementary material for: Mental health disorders among children with special health needs: A population-based cohort study using linked administrative data from Manitoba, Canada
Source: PLoS One. 2025 Jun 25;20(6):e0326672. doi: 10.1371/journal.pone.0326672 (PMC12194185; doi:10.1371/journal.pone.0326672)
Supplement: S6 Table — Manitoba children in 2006, 2007, 2009, and 2011. Odds ratios and 95% confidence intervals. (DOCX) [file pone.0326672.s006.docx]

| **S6 Table. Association between being designated special health needs and receiving a mental health disorder diagnosis by age 16 years. Manitoba children in 2006, 2007, 2009, and 2011. Odds ratios and 95% confidence intervals.** | | | | | |
| --- | --- | --- | --- | --- | --- |
| **Unadjusted** | **N** | **%** | **Odds Ratio** | **95% CI** | **Pr > Chi-Square** |
| **Special Needs in Kindergarten** | 524 | 21.7 | 1.75 | 1.53, 2.01 | <.0001 |
| **Physical Impairment** | 83 | 3.4 | 0.93 | 0.70, 1.23 | 0.6249 |
| **Vision Impairment** | 78 | 3.2 | 0.84 | 0.63, 1.12 | 0.2288 |
| **Hearing Impairment** | 79 | 3.3 | 0.77 | 0.58, 1.02 | 0.069 |
| **Learning Impairment** | 433 | 18.0 | 1.61 | 1.39, 1.86 | <.0001 |
| **Speech Impairment** | 698 | 29.0 | 0.67 | 0.60, 0.74 | <.0001 |
| **Behavioural Impairment** | 813 | 33.7 | 3.27 | 2.87, 3.72 | <.0001 |
| **Emotional Impairment** | 516 | 21.4 | 2.01 | 1.75, 2.32 | <.0001 |
| **Teacher-Reported Need for Further Assessment** | 1704 | 70.7 | 1.35 | 1.21, 1.51 | <.0001 |
| **2+ Categories** | 1291 | 53.6 | 1.67 | 1.50, 1.85 | <.0001 |
|  | | | | |  |
| **Adjusted*** | **N** | **%** | **Odds Ratio** | **95% CI** | **Pr > Chi-**  **Square** |
| **Special Needs in Kindergarten** | 519 | 21.7 | 1.71 | 1.49, 1.97 | <.0001 |
| **Learning Impairment** | 426 | 17.8 | 1.56 | 1.35, 1.81 | <.0001 |
| **Speech Impairment** | 692 | 28.9 | 0.66 | 0.59, 0.74 | <.0001 |
| **Behavioural Impairment** | 806 | 33.7 | 3.21 | 2.81, 3.65 | <.0001 |
| **Emotional Impairment** | 511 | 21.4 | 1.99 | 1.72, 2.29 | <.0001 |
| **Teacher-Reported Need for Further Assessment** | 1691 | 70.7 | 1.32 | 1.18, 1.48 | <.0001 |
| **2+ Categories** | 1280 | 53.5 | 1.62 | 1.46, 1.80 | <.0001 |
| *Adjusted for age below mean at EDI assessment, sex and income quintile. | | | | | |
